# Dietary phytochemicals, HDAC inhibition, and DNA damage/repair defects in cancer cells

**DOI:** 10.1186/1868-7083-3-4

**Published:** 2011-10-26

**Authors:** Praveen Rajendran, Emily Ho, David E Williams, Roderick H Dashwood

**Affiliations:** 1 Cancer Chemoprotection Program, Linus Pauling Institute, 307 Linus Pauling Science Center, Oregon State University, Corvallis OR 97331, USA

**Keywords:** Epigenetics, histone, HDAC, DNA damage, DNA repair, phytochemical, cancer

## Abstract

Genomic instability is a common feature of cancer etiology. This provides an avenue for therapeutic intervention, since cancer cells are more susceptible than normal cells to DNA damaging agents. However, there is growing evidence that the epigenetic mechanisms that impact DNA methylation and histone status also contribute to genomic instability. The DNA damage response, for example, is modulated by the acetylation status of histone and non-histone proteins, and by the opposing activities of histone acetyltransferase and histone deacetylase (HDAC) enzymes. Many HDACs overexpressed in cancer cells have been implicated in protecting such cells from genotoxic insults. Thus, HDAC inhibitors, in addition to unsilencing tumor suppressor genes, also can silence DNA repair pathways, inactivate non-histone proteins that are required for DNA stability, and induce reactive oxygen species and DNA double-strand breaks. This review summarizes how dietary phytochemicals that affect the epigenome also can trigger DNA damage and repair mechanisms. Where such data is available, examples are cited from studies *in vitro *and *in vivo *of polyphenols, organosulfur/organoselenium compounds, indoles, sesquiterpene lactones, and miscellaneous agents such as anacardic acid. Finally, by virtue of their genetic and epigenetic mechanisms, cancer chemopreventive agents are being redefined as chemo- or radio-sensitizers. A sustained DNA damage response coupled with insufficient repair may be a pivotal mechanism for apoptosis induction in cancer cells exposed to dietary phytochemicals. Future research, including appropriate clinical investigation, should clarify these emerging concepts in the context of both genetic and epigenetic mechanisms dysregulated in cancer, and the pros and cons of specific dietary intervention strategies.

## Introduction

Genomic instability is a key feature of cancer development, often associated with the acquisition of mutations in oncogenes, tumor suppressor genes, and DNA repair genes [1]. Thus, DNA repair pathways and cell cycle checkpoint controls have important consequences for genome stability, and have come under much scrutiny [2]. Defects in genome stability increase the sensitivity of cells to DNA damaging agents, and provide an "Achilles heel" for cancer therapeutics [3]. Indeed, there are numerous efforts to manipulate the DNA damage response so as to selectively induce tumor cell death through catastrophic genomic instability [4,5]. Differences in the DNA damage response between normal cells and cancer cells often underlie the utility of DNA damaging agents in cancer treatment. Radiotherapy and chemotherapeutic drugs are known to function by DNA damage-induced tumor cell death, and there are ongoing efforts to improve sensitivity while overcoming resistance to these agents. Poly(ADP-ribose)polymerase (PARP) inhibitors that target defects in double-strand break repair in women with hereditary breast cancer [6] illustrate the concept of selective "synthetic lethality". Other examples include inhibitors of apurinic/apyrimidinic endonuclease-1 (APE1), DNA repair protein RecA homolog (RAD51), ataxia-telangiectasia mutated (ATM), and DNA-dependent protein kinase (DNAPK), some of which have entered clinical trials. As we learn more about the DNA damage response pathways dysregulated in cancer cells, new combinations of agents are being developed with enhanced therapeutic efficacy [7].

Epigenetic mechanisms influence DNA damage and repair pathways; the reader is referred to related reviews in the current journal [8-10]. In eukaryotic cells, DNA damage repair occurs in the context of chromatin, and it is now clear that DNA damage response impacts specific aspects of chromatin structure and chromatin remodeling. Post-translational histone modifications, histone variants, and chromatin-binding proteins provide a regulatory platform for controlling DNA template-directed processes, including gene transcription, DNA replication, and repair of DNA damage [11,12]. Such responses may be mediated by chromatin modifiers involved in histone methylation, acetylation and biotinylation [13-15].

Recently, it was reported that histone deacetylase (HDAC) inhibitors have the potential to interfere with DNA repair mechanisms [16]. A recent review summarized the ways in which HDAC inhibitors trigger apoptosis by taking advantage of genomic instability in cancer cells [14]. The latter review highlighted the ways in which HDAC inhibitors lead to impaired mitotic progression, defects in kinetochore assembly, and aberrations in spindle assembly checkpoint controls, resulting in premature exit from mitosis. HDAC inhibitors regulate chromatin structure and activate the DNA damage checkpoint pathway involving ATM [17]. Histone acetyltransferase (HAT) inhibition also has been shown to impair double-strand break repair [18]. Damage signaling involves phosphorylation of H2AX(S139) (γH2AX) by ATM/ATR kinases. This is followed by chromatin opening and the involvement of H3/H4 acetylation, via HATs such as Tip60, GCN5 and CBP/p300. Chromatin restoration after repair involves γH2AX dephosphorylation by phosphatases PP4 and PP2A and deacetylation of H3/H4 lysines by HDACs. Additional histone modifications such as ubiquitination and sumoylation of histones also contribute to this process. Details of this process have been extensively reviewed elsewhere [19] and dealt with in more detail in the next section.

Similarly, acetylation of non-histone proteins can influence chromatin dynamics, protein turnover, and the DNA damage response. Robert et al [20] have recently shown in yeast that depletion of class I and II HDACs by mutation, or via HDAC inhibition with valproic acid (VPA), prevented DNA damage signaling and interfered with DNA break repair. The DNA resection and recombination protein Sae2 (human C-terminal binding protein interacting protein, CtIP) was acetylated, resulting in increased protein turnover and degradation by autophagy. Deacetylation by HDACs stabilized Sae2, but VPA inhibited this process [20]. Consistent with these observations, a recent study showed that a class III HDAC (SIRT6) positively regulated the repair of double-strand breaks (DSBs) through deacetylation of CtIP [21].

The investigation of genome stability and epigenetics dovetails with mechanistic studies on diet and nutrition. Based on epidemiological studies, diets rich in fruits and vegetables can offer protection against cancer development [22-25]. Recent reviews have covered the mechanisms of dietary agents impacting DNA methyltransferases, HDAC or HAT enzymes, and microRNAs [26-29]. In the context of DNA damage, a folate/methyl deficient diet has been conclusively shown to cause genomic instability [30]. Although dietary anticancer compounds modulate drug metabolizing enzymes and scavenge free radicals, under some conditions they have been shown to generate reactive oxygen species (ROS) and cause oxidative DNA damage [31,32].

Given this background, the present review summarizes recent advances in our understanding of HDACs involved in the DNA damage response, and the possible implications for cancer therapy. Targeting genome integrity in rapidly cycling cells has been a central feature of cancer therapeutics. However, a growing area of interest is the dietary agents that can trigger a DNA damage response via epigenetic mechanisms, involving altered HDAC/HAT activities.

## Changes in chromatin structure during DNA damage

**Figure 1:**
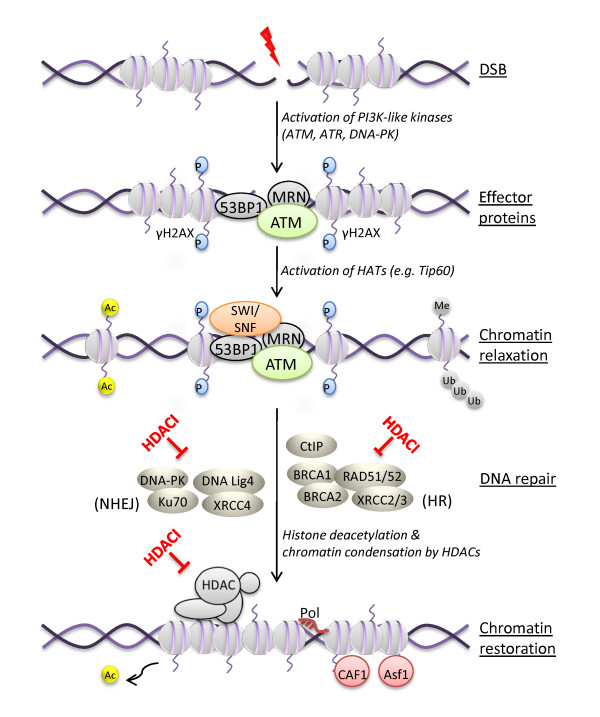
**Model of DNA damage signaling, histone acetylation and chromatin remodeling**. Recognition and signaling of a DSB is followed by opening of chromatin to repair the break, terminating in chromatin restoration after DNA break repair. HDAC inhibition, as indicated by HDACI, has been shown to affect key steps in this process (as illustrated) by virtue of deacetylating histone and non-histone proteins involved in signaling and repair.

DNA wraps around an octameric complex of core histones H2A, H2B, H3 and H4 to form nucleosomes. DSBs induced by ROS, replication stress, or by exogenous agents like UV, radiation, radiotherapy, or other genotoxic agents are thought to be the most dangerous lesions for genomic integrity [33]. Although the exact sequence of events following DSB is still poorly understood, one of the earliest events in the response to DNA breakage involves phosphorylation of H2AX (γH2AX) that surrounds ~2 Mb of each DSB, which marks the sites of breakage [34,35]. Thus, a common biomarker for DNA-damage nuclear foci is γH2AX, typically assayed by immunofluorescence-based approaches. H2AX is phosphorylated by phosphatidylinositol-3 kinase (PI3K)-like kinases, including ATM, ATM-and Rad3-related (ATR), ATM related kinase (ATX), and DNAPK [36,37]. Histone H3 acetylation at tail residues K9, K14, K18, K23 and K27, and histone H4 acetylation at tail residues K5, K8, K12, K16 [38] reduces their affinity for negatively-charged DNA. This in turn promotes relaxation of chromatin, and facilitates access of repair proteins. The HAT complex TAT-interacting protein 60 (TIP60) acetylates histones H2A, H3, and H4 [39,40], whereas HDACs participate in histone deacetylation during repair and chromatin reassembly [41-43], as shown in Figure 1.

H2AX phosphorylation and core histone acetylation assist in the recruitment to DSB sites of chromatin remodeling complexes of the SWI2/SNF2 superfamily [44-46]. This is followed by the accumulation of other PI3K-like members, mediator of DNA damage checkpoint protein 1 (MDC1) or p53-binding protein (53BP1), which play a pivotal role in signaling DSBs [47]. DSB signaling is further amplified by transducer checkpoint kinases, CHK1 and CHK2, which, together with ATM and ATR, phosphorylate breast cancer 1 (BRCA1), RAD51, p53, and its negative regulator, murine double minute (Mdm2) [48]. Phosphorylation of p53 leads to its stabilization, causing cell cycle arrest through induction of cyclin-dependent kinase inhibitor p21 or in the event of severe DNA damage, apoptosis.

DNA damage is sensed and the repair machinery is employed, consisting of MRE11-RAD50-Nbs1 (MRN) mediator complexes or RAD51 enzymes [49,50] that recruit ATM to the site of DSBs [51]. Histone ubiquitylation, through ubiquitin ligases RNF8 and 168, is an important route for recruitment of additional repair complexes involving BRCA1/Abraxas/Rap80 [52,53].

Other events include mobilization of high-mobility group N1 (HMGN1) protein for ATM recruitment, and heterochromatin protein 1β (HP1β) [54]. The histone trimethylation mark, H3K9me3, is recognized by chromodomain regions of HP1 and casein kinase 2 (CK2) that mediate the removal of HP1 protein [55]. The recruitment and activation of ATM at DSB sites affects chromatin structure by phosphorylating the KRAB associated protein (KAP-1), thus further relaxing the chromatin structure [56]. Acetylation of histone H3K56 drives chromatin assembly after repair, and signals the completion of repair [57].

The mechanisms that restore chromatin architecture after repair of DSBs involve deacetylation by HDACs [58], proteasomal degradation of MDC1 foci [59,60], and turnover of the repair machinery. Chromatin assembly factors, including the histone chaperones chromatin assembly factor I (CAF-1) and anti-silencing function 1(Asf1), play essential roles in restoring chromatin structure and cell cycle progression after DNA repair [57].

## Role of HDACs in DNA damage response

Acetylation is a reversible process in which histone and non-histone protein acetyltransferases transfer the acetyl moiety from acetyl co-enzyme A to lysine residues, and HDACs remove the acetyl groups. HDACs play major roles in modulating chromatin accessibility during transcription, replication, recombination, and repair [61,62]; however, the role of individual HDACs in these processes is still unclear.

At the present time, 18 HDACs have been identified in humans that fall into four classes: class I HDACs (HDAC1, 2, 3 and 8) share sequence similarity with the yeast RPD3 deacetylase, are ubiquitously expressed, and they are localized mainly in the nucleus. Class II HDACs (HDAC4, 5, 6, 7, 9 and 10) are homologous to the yeast Hda1 deacetylase, are nuclear and cytoplasmic, and restricted to certain tissues. Class II HDACs are further subdivided into class IIa (HDAC4, 5, 7 and 9) and class IIb (HDAC6 and 10). Class III HDACs are represented by sirtuins (SIRT1 to SIRT7), a family of seven HDACs sharing homology with yeast silent information regulator 2 (Sir2). Class IV has only one member, HDAC11, which shares conserved residues with both class I and II HDACs [63]. Class I, II, and III HDACs have been implicated in the DNA damage response, homologous recombination (HR), and chromatin integrity. This is explained below, and summarized in Table 1.

**Table 1:** HDACs implicated in chromatin structure/function during DNA damage and repair

HDAC	Role in DNA damage/repair	Substrates involved in DNA damage response	References
***Class I***			

HDAC1	Protects from DNA damage, sustains DNA damage checkpoint, maintains DNA replication, regulates oxidative stress and NHEJ	H3K56, p21, p53, BRCA1, CHES1, PCNA, Top II, ATM, ATR, RFC, ING1a, APE1/Ref1	[41,64-71,321]

HDAC2	Participates in DNA damage signaling by translocation to nucleus; regulates DNA repair	H3K56, BRCA1, ATR	[41,66,68]

HDAC3	Protects from DNA damage, maintains replication fork, mitotic spindle and helps in DNA repair and genomic stability via HDAC3/NCOR/SMRT complexes	H3K9/K14, H4K5/K12	[42,72,75-77]

***Class IIa***			

HDAC4	Increases DNA repair by translocation to the nucleus and signaling repair	53BP1	[78,79]

HDAC9	DNA repair through homologous recombination	Not yet identified	[43]

***Class IIb***			

HDAC6	Role in chemosensitization	GADD153	[80]

HDAC10	DNA repair through homologous recombination	Not yet identified	[43]

***Class III***			

SIRT1	Protects from oxidative DNA damage, maintains telomere length and activates DNA repair through HR, NER, and BER	p53, FoXO1, WRN, Ku70, Tip60, APE1, H3K56, NBS1, MRN, telomere, XPA, XPC	[81-83,86-94,97,98]

SIRT3	Transports to mitochondria and reduces oxidative DNA damage	Idh2, H4K16	[99,100]

SIRT6	Promotes DNA repair by HR, forms a complex with DNA-PK and resists DNA damage; maintains chromatin structure and genomic stability	H3K9/K56, CtIP, XPA, DNAPK	[21,101,102]

*Information regarding HDAC 5, 7, 8 and 11 is currently lacking in terms of a definite role in this mechanism*.

An important substrate of HDAC1 is the tumor suppressor protein p53. Recruitment of HDAC1 by MDM2 promotes p53 degradation by deacetylation. Thus, HDAC1 decreases DNA damage-induced p53 acetylation, and inhibits the induction of p21 and MDM2 [64]. HDAC1 also regulates several other proteins involved in the DNA damage response, such as proliferating cell nuclear antigen (PCNA) [65], BRCA1 [66], ATM [67], ATR [68], inhibitor of growth 1a (ING1a) [69], replication factor C (RFC) [70], apurinic apyrimidinic endonuclease redox effector factor-1 (APE1/Ref1) [71], and proteins that facilitate non-homologous end-joining (NHEJ) by altering histone H3K56 acetylation [41].

Miller et al. [41] showed that HDAC1 and HDAC2 cooperate in the DNA damage response. Specifically, HDAC1 and HDAC2 were recruited to DNA damage sites and regulated the deacetylation of H3K56 and H4K16, a requirement for DNA repair, particularly through NHEJ. HDAC2 also regulates ATR [68], and alters histone H3K56 acetylation status during the DNA damage response. Based on their findings, the authors suggested that HDAC1 and HDAC2 might repress transcription at sites of DNA damage, thereby preventing transcription from interfering with repair processes, as well as remodeling chromatin into a state that promotes NHEJ. They found that Class I/II HDAC inhibitors, such as butyrate and trichostatin A (TSA), caused defects in the DNA damage response, including hyperacetylation of H3K56 and H4K16, and impairment of NHEJ. Furthermore, HDAC1- and 2-depleted cells were hypersensitive to DNA-damaging agents and showed sustained DNA-damage signaling, phenotypes that reflect defective DSB repair. The authors discussed the potential implications of their findings for HDAC1- and HDAC2-specific therapy [41].

Bhaskara et al. [42,72] showed that HDAC3 is important for DSB repair. HDAC3 associates with nuclear receptor corepressor (NCOR) and silencing mediator for retinoic and thyroid receptor (SMRT) [73], and is considered a locus-specific corepressor that is recruited to promoters to repress genes regulated by nuclear hormone receptors and other transcription factors [74]. Conditional deletion demonstrated the absolute requirement for cell viability of HDAC3 in murine embryonic fibroblasts (MEFs) [72]. The latter MEFs underwent apoptosis due to impaired S phase progression and formation of DSBs, rather than altered transcriptional programs. The DNA damage was blocked when cells were taken out of the cell cycle by serum starvation, suggesting that HDAC3 acted during S phase. In another study [42], HDAC3-null MEFs increased histone acetylation (H3K9, H3K14, H4K5 and H4K12) in late S phase. Knockdown of NCOR1 and SMRT increased acetylated H4K5 and caused DNA damage, indicating that the HDAC3/NCOR/SMRT axis may be critical for maintaining chromatin structure and genomic stability. Furthermore, two studies have linked HDAC3 to maintenance of the mitotic spindle assembly [75,76]. Ishii et al. [75] reported on the localization of HDAC3 to the mitotic spindle, and showed that HDAC3 knockdown led to chromosome misalignment, impaired kinetochore-microtubule attachment, and mitotic spindle collapse. Eot-Houllier et al. [76] showed that HDAC3 knockdown induced spindle assembly checkpoint activation and sister chromatid dissociation. Further, down-regulation of HDAC3 mimics actions of the HDAC inhibitor suberoylanilide hydroxamic acid (SAHA, vorinostat) in reducing replication fork velocity and increasing origin firing at sites of replication, likely due to chromatin changes [77].

Among the class II HDACs, HDAC4, and more recently HDAC6, HDAC9 and HDAC10, have been implicated in DNA damage signaling, transcription factor binding, and DNA repair processes [78-80,43]. Kao et al. [78] showed that HDAC4 co-localized with 53BP1, a PI3K-like member with a pivotal role in signaling DSBs. HDAC4-containing foci gradually disappeared in repair-proficient cells, but persisted in repair-deficient cell lines, suggesting that resolution of HDAC4 foci is linked to successful DNA repair. Silencing of HDAC4 via RNA interference surprisingly also decreased levels of 53BP1 protein, abrogated the DNA damage-induced G2 delay, and radiosensitized HeLa cells. These observations showed that HDAC4 is a critical component of the DNA damage response pathway that acts through 53BP1, and perhaps contributes in maintaining the G2 cell cycle checkpoint. Basile et al. [79] demonstrated that HDAC4 shuttles from the cytoplasm to the nucleus following DNA damage, independent of p53 activation, and becomes associated with gene promoters via a p53-dependent mechanism. Thus, HDAC4 is clearly implicated as a component of the DNA damage response.

Namdar et al. [80] reported that HDAC6 inhibition with tubacin or shRNA activated the intrinsic apoptosis pathway in cancer cells; this led to accumulation of γH2AX, and the expression of growth arrest and DNA damage 153 (GADD153/DDIT3), a transcription factor upregulated in response to cellular stress. Tubacin treatment enhanced cell death induced by topoisomerase II inhibitors etoposide and doxorubicin, and by the pan-HDAC inhibitor SAHA, in transformed cells (LNCaP, MCF-7), an effect not observed in normal cells (human foreskin fibroblast cells). Further, tubacin increased the accumulation of γH2AX and activated Chk2. GADD153/DDIT3 induction was augmented when tubacin was combined with SAHA. The authors suggested that HDAC6-selective inhibition enhances the efficacy of certain anticancer agents in transformed cells [80].

Recently, Kotian et al. [43] showed that depletion of HDAC9 or HDAC10 inhibited HR in a tissue-culture based homology-directed repair assay. The authors showed that HDAC9 and HDAC10 were directly involved in the HR process, and this was not through indirect blocking of the cell cycle. Further, depletion of HDAC9 or HDAC10 resulted in increased sensitivity to mitomycin C [43].

Among the NAD^+^-dependent class III HDACs (sirtuins), SIRT1, SIRT3 and SIRT6 have definite roles in genome stability and repair [21,81-84]. SIRT1 plays crucial roles in multiple biological processes affecting gene transcription, cellular metabolism, stress response, and tumorigenesis. SIRT1 is overexpressed in several p53-deficient tumor cell lines, and the transient knockdown of SIRT1 leads to increased apoptosis after DNA damage or oxidative stress [85]. Moreover, several proteins involved in the DNA damage response are deacetylated and inactivated by SIRT1. These targets include p53 [86,87], forkhead box transcription factor (FoxO) [88,89], the nonhomologous end joining (NHEJ) factor, Ku70 [90], Tip60 [91], the histone modification H3K56 acetylation [92], and MRN repair complex [93]. Thus, these studies support the idea that SIRT1 can act as an oncogenic protein when overexpressed in cancer cells.

SIRT1 also is thought to act as a tumor suppressor in some scenarios, through its role in deacetylating p53 [94] and Ku70 [90]. CK2 phosphorylates and activates SIRT1, and partly protects cells from ionizing radiation-induced apoptosis [95], whereas Set7/9 methylates SIRT1 and disrupts it's binding to p53, leading to p53 acetylation and activation in response to DNA damage [96]. Several recent reports have shown the importance of SIRT1 in enhancing DNA repair [81-83,97]. Palacios et al. [81] examined the effects of SIRT1 on telomere maintenance and DNA repair. Using SIRT1-deficient and gain-of-function mouse models, SIRT1 was identified as a positive regulator of telomere length *in vivo*, and attenuated telomere shortening associated with aging. The authors showed that SIRT1 interacted with telomeric repeats *in vivo*. In addition, SIRT1 overexpression increased HR throughout the entire genome, including telomeres, centromeres, and chromosome arms. These findings link SIRT1 to telomere biology and global DNA repair, and provide new mechanistic insights into the known functions of SIRT1 in the protection from DNA damage [81]. Uhl et al. [82] showed that Werner helicase (WRN) was required for SIRT1-mediated HR. WRN, in its mutated form, causes premature aging and cancer, and has been linked to Rad51-independent single-strand annealing (SSA) DSB repair pathway. SIRT1 also regulates other DNA repair pathways, *viz*. base-excision repair (BER) and nucleotide-excision repair (NER) [83,97,98] through the transcription of xeroderma pigmentosum (XPA, XPC) group proteins [97,98]. Yamamori et al. [83] showed that SIRT1 plays a vital role in maintaining genomic integrity by deacetylating APE1, which is an essential component of the BER pathway. Increased association of SIRT1 with APE1 during genotoxic stress facilitated SIRT1-mediated deacetylation of APE1 *in vitro *and *in vivo*, thereby reducing genotoxic insult-stimulated lysine acetylation of APE1. Fan and Luo [97] showed that SIRT1 plays an important role in the regulation of NER. Thus, downregulation of SIRT1 significantly sensitized cells to UV irradiation through interaction with xeroderma pigmentosum group A (XPA), a core factor essential for NER. SIRT1 has been shown to deacetylate XPA both *in vitro *and *in vivo *[97].

SIRT3 is transported from the nucleus to the mitochondria upon cellular stress, as in the case of DNA damaging agents, and deacetylates histone H4K16 [99]. SIRT3 has been shown to deacetylate and activate mitochondrial isocitrate dehydrogenase 2 (Idh2), leading to increased NADPH levels and an increased glutathione GSH:GSSG ratio in mitochondria, thereby protecting cells from oxidative stress-induced cell death. SIRT3 is thus an essential player in the mitochondrial glutathione antioxidant defense system [100].

Kaidi et al. [21] have shown that human SIRT6 has a role in promoting DNA end-resection, a crucial step in DSB repair by HR. SIRT6 depletion impaired the accumulation of replication protein A (RPA) and single-stranded DNA at damage sites, reduced the rate of HR, and sensitized cells to DSB-inducing agents. The authors identified CtIP as a SIRT6 interaction partner, and showed that SIRT6-dependent CtIP deacetylation promotes DSB resection. Schwer et al. [101] have shown that SIRT6 deletion causes hyperacetylated histone H3K9 and H3K56, two chromatin marks implicated in the regulation of gene activity and chromatin structure, in various brain regions. McCord et al. [102] observed that SIRT6 forms a complex with DNAPK and promotes DSB repair. In addition, the role of SIRT6 in genomic stability has been demonstrated in aging mouse models [84].

Collectively, these studies highlight the roles of multiple HDACs in the DNA damage response and chromatin stability. As a corollary, the question arises as to how such events might be impacted by HDAC inhibitors.

## HDAC inhibitors and the DNA damage response

HDAC inhibitors are being developed as anticancer agents, as well as therapies for non-oncologic disorders [63,103]. Inhibitors of the zinc-dependent HDACs belong to several chemical classes, including hydroxamic acids, cyclic peptides, electrophilic ketones, short-chain fatty acids, and benzamides. Some of these inhibitors affect the interactions of HDACs with protein partners, independent of the deacetylase activity [63]. Thus, HDAC inhibitor mechanisms now include competitive binding in the active site [104], turnover of the HDAC protein by proteasomal degradation [105], and HDAC protein inactivation by alkylation/carbonylation [106,107]. These HDAC regulatory mechanisms are not necessarily mutually exclusive.

HDAC inhibitors can induce growth arrest of neoplastically-transformed cells and trigger apoptosis via one or more pathways. These events are associated with altered patterns of acetylation in histone and non-histone proteins, including key players involved in the regulation of gene expression, apoptosis, cell cycle progression, redox signaling, mitotic division, DNA repair, cell migration, and angiogenesis [63]. Subsequent to the role of HDACs in maintaining genome stability, as discussed above, histone hyperacetylation induced by HDAC inhibitors causes structural alterations in chromatin. This can open up regions of DNA that are normally protected by heterochromatin, enabling DNA-damaging agents to gain access to the exposed template. Importantly, HDAC inhibitors have been shown to decrease the expression of DNA repair proteins such as Ku70 [108], BRCA1 [109], RAD51 [110] and CtIP [20]. It is not clear whether transcription mediates HDAC inhibitor actions in these circumstances [111], and indeed non-transcriptional targets of HDAC inhibitors have been proposed [112,113]. Thus, HDAC inhibitors have the potential to target multiple signaling and repair mechanisms in the DNA damage pathway by targeting histones and non-histone proteins, as illustrated in Figure 1.

Several pharmacologic HDAC inhibitors are undergoing clinical trials as monotherapies, or in combination therapies with other anticancer agents. Two of these HDAC inhibitors, vorinostat and romidepsin (depsipeptide), have been approved for the treatment of cutaneous T-cell lymphoma [114]. Apart from effects on gene transcription, evidence is accumulating that HDAC inhibitors influence chromatin stability, mitosis, and DNA repair mechanisms. For example, vorinostat acts at replication origins [77], downregulates the DNA repair gene *Rad52 *[115], and suppresses HR repair genes such as Brca1, Rad51, Chk1, and Bubr1 (a checkpoint kinase), via downregulation of E2F1 transcription factor [16]. These effects also have been reported for other HDAC inhibitors, such as PCI-24781 [110] and VPA [16]. Romidepsin downregulated thioredoxin reductase (TrxR), generated ROS accumulation, and augmented DNA damage and apoptosis [116]. The HDAC inhibitor LAQ-824 also triggered ROS production, with increased γH2AX and Ku70 acetylation [117]. Many other HDAC inhibitors, including TSA, SAHA and MS-275, augment the acetylation of Ku70 [108] and alter genes encoding HR components, such as *ATR*, Bloom syndrome gene (*BLM*), *BRCA1, BRCA2*, and nijmegen breakage syndrome 1(*NBS1*) [109].

## Dietary agents and their effects on chromatin, DNA damage, and repair in cancer cells

**Figure 2:**
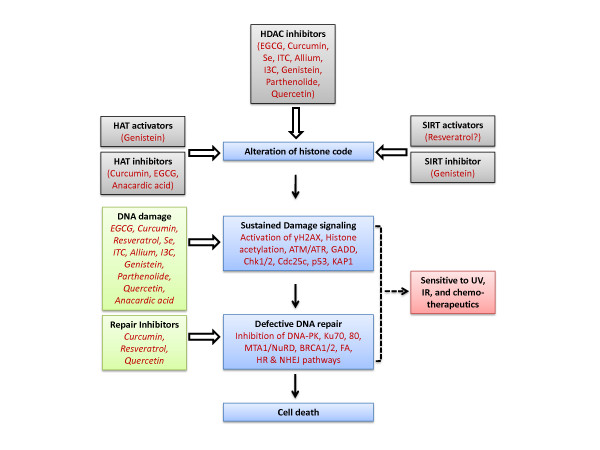
**The role of dietary factors in altering histone acetylation and DNA damage signaling**.

In addition to the potent HDAC inhibitor drugs being developed as cancer therapeutic agents, there is growing interest in dietary phytochemicals that also possess HDAC inhibitor activity [118,119,26]. A synopsis of dietary chemopreventive agents in the context of DNA damage and repair pathways is shown in Figure 2, and is summarized below for specific chemical classes. As discussed next, some dietary compounds have shown DNA-damaging effects in cancer cells associated with HDAC inhibition. The order in which the compounds are presented below corresponds with the approximate extent of supportive evidence from the literature for HDAC/HAT modulation and DNA damage end-points.

### Isothiocyanates

Brassica or cruciferous vegetables are a rich source of glucosinolates [120]. The hydrolysis of these glucosinolates by the plant enzyme myrosinase generates biologically active isothiocyanates (ITC) and indoles [121]. For example, ITC precursors of sulforaphane (SFN) and phenethyl isothiocyanate (PEITC) are found at high levels in broccoli and watercress, respectively. Epigenetic effects of ITCs have been linked to the inhibition of HDAC activity and histone hyperacetylation, as reported for SFN [122], allyl isothiocyanate (allyl-ITC) [123], benzyl isothiocyanate (BITC) [124], phenylhexyl isothiocyanate (PHITC) [125], PEITC [126], and other longer-chain isothiocyanates [118]. In addition to altering HDAC expression and causing histone acetylation, other histone marks altered by ITCs include histone methylation [127]. BITC [124] and SFN [128,129] have also been shown to decrease HDAC protein expression in cancer cells.

We know from previous studies that methyl isothiocyanate [130], BITC [131], allyl-ITC and PEITC [132] exert genotoxic effects. For example, BITC (10 μM) increased γH2AX and triggered apoptosis in Capan-2 pancreatic cells [133]. It caused a significant decrease in the expression and activity of HDAC1 and HDAC3, as well as NFκB inactivation, in pancreatic cancer cells but not normal cells. Interestingly, overexpression of HDAC1 or HDAC3 blocked these effects [124].

SFN has been shown to cause both DSBs and single-strand breaks (SSBs) in cancer cells. Sekine-Suzuki et al. [134] observed that 20 μM SFN triggered cell cycle arrest, induced DSB, and elevated γH2AX levels in cervical cancer (HeLa) cells. DSBs generated by SFN were comparable to that triggered with 12 Gy of *X*-rays. These DSBs were repaired mainly by HR through Rad51 foci formation and not by NHEJ [134]. Sestili et al. [135] reported that a short exposure of cells with SFN (10-30 μM for 1-3 h) triggered SSBs in Jurkat lymphoma and HUVEC cells. They found that DNA damage was causally linked to ROS generation and GSH depletion [135]. DSBs also were triggered in colon cancer cell lines SW620 at 10-50 μM [136] and HCT116 cells at 15 μM SFN, resulting in sustained γH2AX expression (our unpublished data). In prostate cancer cells, SFN-induced DNA damage involved the Chk2-mediated phosphorylation of protein phosphatase Cdc25C [137].

We recently reported SFN-induced loss of HDAC3 and HDAC6 protein expression in a time-dependent manner in HCT116 colon cancer cells, leading to acetylation of histone H4 and tubulin, respectively. By 6 h, SFN was shown to enhance CK2/HDAC3 binding, leading to HDAC3 phosphorylation and nuclear export by 14-3-3 and Pin1 [128]. As noted earlier, this has the potential to affect chromatin structure and DNA repair since HDAC3 is critical for chromatin integrity, mitotic spindle assembly, and DNA replication [72,75,76]. We also found that overexpression of HDAC3 or HDAC6 blocked SFN-induced acetylation of respective substrates [128]. It is interesting to note that prostate [129] and colon [138] cancer cells were more sensitive to SFN as compared to normal cells. Clarke et al. [129] demonstrated differential effects of SFN in normal prostate cells versus hyperplastic and cancerous prostate cells based, at least in part, on altered HDAC expression levels.

Further, ITC-induced oxidative DNA damage has been attributed to ROS generation [139-142], inhibition of telomerase [143], lipid peroxidation [144], and covalent binding to protein targets such as tubulin [145]. Thus, it appears that SFN preferentially targets cancer cells over normal cells possibly *via *a sustained DNA damage response.

### Indole-3-carbinol (I3C) and 3,3'-diindolylmethane (DIM)

Cruciferous vegetables contain glucosinolates such as glucoraphanin, the precursor of SFN, and glucobrassicin, the precursor of indole-3-carbinol (I3C). The latter compound and its acid condensation products, such as 3,3'-diindolylmethane (DIM), have been examined extensively for their cancer chemoprotective properties [146]. I3C has been shown to increase ATM signaling and p53 phosphorylation leading to p21 induction and G_1 _arrest in breast cancer cells [147]. I3C-induced activation of ATM-Chk2 was further shown to degrade the protein phosphatase Cdc25A [148]. Bhatnagar et al. [149] reported that DIM inhibited expression of HDAC1, HDAC2 and HDAC3 in colon cancer cells, which was associated with inhibition of survivin. Li et al. [150] demonstrated that DIM-induced HDAC depletion involved proteasome-mediated HDAC protein degradation. Although the authors found negligible increases in the acetylation of gene promoters, a reduction in the levels of repressive HDACs bound to the p21 and p27 promoters coincided with cell cycle arrest. Further, DIM caused significant increases in γH2AX and chromatin relaxation, with phosphorylation of KAP-1 prior to DNA damage-triggered apoptosis. Interestingly, decreased HDAC expression appeared 24 h prior to DNA damage signaling, suggesting HDAC inhibition/loss as a possible causative mechanism [150].

Other mechanisms related to the DIM-induced DNA damage response include activation of BRCA1 in breast and prostate cancer cells. BRCA1/2 signaling by DIM led to endoplasmic reticulum stress and activation of the *GADD45 *promoter [151]. Similarly, another study demonstrated that I3C, in combination with genistein, induced *GADD *gene expression in MCF-7 breast cancer cells and decreased expression of ER-α, thereby triggering apoptosis [152].

### Parthenolide

Parthenolide (PN) is a sesquiterpene lactone isolated from *Tanacetum parthenium*. It has been shown to cause cell cycle arrest, promote cell differentiation, and induce apoptosis [153]. In addition to its other actions, PN was found to specifically deplete HDAC1 protein without affecting other class I/II HDACs. HDAC1 depletion was found to occur via proteasomal degradation that was activated through the DNA-damage-transducer ATM [154]. HDAC1 depletion by PN led to ubiquitination of MDM2 leading to p53 activation and sustained DNA damage response [155].

### Anacardic acid

A phytochemical that modifies DNA damage *via *HAT inhibition is anacardic acid. The anacardic acid 6-pentadecyl salicylic acid (6-PDSA), from cashew nut shell liquid, is a potent HAT inhibitor. It inhibits p300 and p300/CBP-associated HAT activities [156]. In addition, 6-PDSA was shown to inhibit the HAT function of Tip60 and sensitize cancer cells to ionizing radiation [157]. Interestingly, a structural analog of 6-PDSA was reported to reduce histone H3K56 acetylation [158]. On the contrary, in normal human dermal fibroblasts, inhibition of HAT activity by 6-PDSA prevented UV-induced increases in γH2AX, p53, and acetyl-H3 [159], suggesting that histone acetylation is a prerequisite for efficient DNA damage signaling in normal cells.

### Allium compounds

Garlic, onions, shallots and other members of the *allium *family contain an interesting and complex range of water-soluble and fat-soluble organosulfur compounds, some of which have been implicated as cancer chemopreventive agents [160,161]. Allyl derivatives from garlic were among the first compounds described to impact histone acetylation status. Allyl mercaptan (AM), diallyl disulfide (DADS), *S*-allylcysteine (SAC), *S*-allylmercaptocysteine (SAMC) and allicin increased histone acetylation (H3/H4) in human cancer cells [123,162-164], implicating HDACs as possible targets. AM was the most effective HDAC inhibitor among several garlic-derived organosulfur compounds and their metabolites, including SAMC, SAC, diallyl sulfide (DAS), DADS, diallyl trisulfide (DATS) and allyl methyl sulfide (AMS). In human colon cancer cells, AM caused histone H3 hyperacetylation, and facilitated Sp3 and p53 binding on the *P21WAF1 *promoter [165].

Recently, DADS and DATS were shown to directly induce the DNA damage response in cancer cells [166,167]. In skin cancer cells, 25 μM DATS increased γH2AX levels as early as 3 h and produced a 10-fold increase in γH2AX by 24 h. Furthermore, DATS increased the phosphorylation of p53 by 12 h, and induced p21 expression at 24 h. Importantly, such effects were noted in cancer cells but not in normal keratinocytes [166]. The authors suggested that DATS might increase ROS levels and inflict DNA damage. A prior study showed that DATS activated the Chk1-Chk2-Cdc25C pathway, causing cell cycle arrest in prostate cancer cells [168]. Ling et al. [167] reported that DADS induced G_2_/M arrest through a similar pathway involving Chk1-Cdc25c-cyclin B1, and the DNA damage signaling kinase ATR. The specific role of histone acetylation in DNA damage signaling has not been elucidated in these studies. However, the ATR signaling pathway, known to be activated by *allium *compounds, is known to initiate a p53 phosphorylation-acetylation cascade leading to p21 expression [169]. In fact, the DNA damage-mediated phosphorylation of p53 promotes acetylation by increasing interaction between p53 and HATs [169]. Whether *allium *compounds affect DNA repair mechanisms in addition to DNA damage is not clear, since one study shows that DADS does not affect DNA repair genes in a microarray-based study using cancer cells [170].

Numerous studies have implicated ROS, NOS, and hydrogen peroxide (H_2_O_2_) in the actions of DADS and DATS, with evidence for anti-cancer activities being blocked by ROS scavengers such as N-acetyl cysteine (NAC) and other anti-oxidants [166,171-174].

### Selenium

Selenium is an essential trace element found as inorganic forms in soil, but also bioaccumulated as organic forms in foods such as Brazil nuts and seafood. Anticarcinogenic effects have been attributed to selenoproteins, and more recently to organoselenium metabolites [175-177]. Selenium may be an effective chemopreventive and anticancer agent in a broad spectrum of human cancers, *viz*. prostate, colon, bladder, lung, liver, ovarian, and leukemia [178]. Some forms of selenium exert epigenetic effects via histone modifications. HDAC activity was decreased, and histone acetylation increased, by sodium selenite [179], keto-methylselenobutyrate (KMSB), methyl selenocysteine (MSC), and methyl selenopyruvate (MSP) [180,181]. Histone phosphorylation also was increased by selenomethionine (SM) on the promoters of *GJB2 *(*connexin 26*) and *serum glucocorticoid kinase *genes [182].

Selenium compounds have been reported to cause DNA damage-mediated apoptosis in cancer cells [183]. Recently, two papers described the mechanisms by which selenium compounds trigger DNA damage-induced cell death in cancer cells but not in normal cells [184,185]. Qi et al. [184] examined methylseleninic acid (MSA, 0-10 μM), methyl selenocysteine (MSC, 0-500 μM), and sodium selenite (0-20 μM) in mismatch repair (MMR)-deficient HCT116 colorectal cancer cells and MMR-proficient HCT116 cells with MutL homolog 1 (MLH1) complementation. The authors found that compared with MMR-deficient HCT116 cells, HCT116+hMLH1 cells were significantly more sensitive to oxidative DNA lesions and γH2AX induction. Further, response to selenium compounds was dependent on ATM kinase and ROS, and required hMLH1-hPMS2. Addition of the ATM kinase inhibitor KU55933, the antioxidant NAC, or the superoxide dismutase mimic Tempo, suppressed the selenium-induced effects. The authors suggested that the hMLH1-hPMS2 complex senses and processes selenium-induced oxidative DNA damage and transmits the signal to ATM kinase, leading to the activation of G2/M checkpoint and death pathways [184]. Hence, in this case, a DNA repair complex acts via genomic instability and mutation to induce cell death. Wu et al. [185] showed that selenium compounds activated similar responses in normal MRC5 cells; however, rather than apoptosis induction they activated cell senescence, as evidenced by the expression of senescence-associated β-galactosidase and BrdU incorporation. In view of the HDAC inhibition, as noted previously for these compounds, it will be interesting to probe whether histone modifications have a role to play in the observed DNA damage signaling. In this regard, we know that MSA and MSC activate ATM [184], which is known to control the transcription of DNA damage genes in response to HDAC inhibition [186]. SM, another selenium compound, also decreased cell proliferation and induced cell-cycle arrest by increasing GADD34 and GADD153 expression [187]. However, such effects were not seen in mammary and prostate cancer cells [188]. Selenocystine, a nutritionally available selenoamino acid, was shown to induce ROS formation leading to DNA strand breaks in cancer cells, but not in normal human fibroblasts [189]. In fact, in normal fibroblast cells, selenium was identified as an important cofactor for various antioxidant enzymes that enhance DNA repair in cells [190].

### Polyphenols

Polyphenols occur naturally in many foods and beverages consumed by humans. Promising cancer chemopreventive polyphenols include those in green tea, curry spices, grapes, soy, and berries.

#### (-)-Epigallocatechin-3-gallate (EGCG)

EGCG, the most abundant polyphenolic catechin in green tea, was identified as an antioxidant *in vitro *[191], although the possible relevance of this activity to its anticancer properties *in vivo *is far from established [29]. EGCG was reported to inhibit enzymes involved in DNA methylation, and was subsequently identified as a histone modifier [192-194]. EGCG inhibited HDAC activity and increased histone acetylation in prostate [192], skin [193], and breast cancer cells [194]. Pandey et al. [192] demonstrated that EGCG reduced mRNA expression of HDAC1, HDAC2, and HDAC3, leading to re-expression of GSTP1 in prostate cancer cells. Li et al. [194] showed that EGCG reactivated estrogen receptor (ERα) in breast cancer cells, due to decreased binding of the transcription repressor complex Rb/p130-E2F4/5-HDAC1-SUV39H1-DNMT1. Interestingly, Choi et al. [195] identified EGCG as a HAT inhibitor that suppressed transcription factor p65 (RelA) acetylation, thereby inhibiting nuclear factor kappa B (NFκB), interleukin 6 (IL6), and downstream target genes. In addition to the HAT and HDAC activities, EGCG inhibited polycomb group (PcG) proteins [196] that are key epigenetic regulators [197]. Treatment of skin cancer cells with EGCG reduced expression of PcG proteins BMI-1 and EZH2, leading to global reduction of histone H3K27me3 and reduced cell survival [196].

Although EGCG exhibits antioxidant activity in some *in vitro *assays, it can induce oxidative DNA damage and generate intracellular and mitochondrial ROS in lung cancer cells [198]. EGCG treatment triggered GADD153 gene expression in combination with celecoxib, via MAPK signaling [199]. Although GADD153 activity is known to be modulated by HDACs [80,200], it is not clear whether HDAC inhibition played a role on *GADD153 *gene activation by EGCG. In this regard, it is worth mentioning that LBH589, a well known HDAC inhibitor, activates *GADD *genes by augmenting histone acetylation at the corresponding gene promoters [201]. More studies need to be carried out to determine if the effects of EGCG on HDACs contribute to its DNA damage effects. Another aspect of EGCG in this pathway is inhibition of CK2 [202], which is an important enzyme in the DNA damage response [55]. In addition, tea catechins are reported to exert DNA demethylating effects *in vitro *[203,204], and trigger oxidative degradation of cellular DNA in the presence of copper Cu(II) ions [205].

#### Curcumin

Curcuminoid polyphenols in Indian spices have antioxidant, anti-inflammatory, and cancer chemoprotective properties [206-209]. There is growing interest in these compounds and their potential to modulate epigenetic endpoints [210-212]. Curcumin, for example, inhibited HAT activity by inducing proteasome-dependent degradation of p300 [213] in multiple cancers at a concentration of 20 μM or higher [214-216]. Curcumin also was shown to inhibit HDAC1 and to upregulate p21 mRNA and protein in a dose- and time-dependent manner in HepG2 hepatoma cells [217]. Another study showed that curcumin inhibited the expression levels of p300, HDAC1, HDAC3, and HDAC8 proteins, repressed NFκB and Notch1, and decreased cell proliferation in Raji lymphoma cells [218]. A more recent report on curcumin also supported its HDAC inhibitory effects [219]. However, curcumin also was found to stabilize HDAC2 protein expression and increase HDAC activity in lung, a beneficial outcome in the context of chronic oxidative stress [220].

Rowe et al. [221] reported that curcumin caused DNA damage in cancer cells, associated with phosphorylation, increased expression, and cytoplasmic retention of the BRCA1 protein. These effects were not seen in normal mammary epithelial cells [221]. Further, the induction of γH2AX and DNA damage by curcumin required ATM/Chk1 signaling [222]. Curcumin induced expression of GADD153 and increased ROS-mediated apoptosis induction in lung cancer cells. Treatment with GADD45- and GADD153-siRNAs inhibited apoptotic induction in these cells [223,224]. As noted earlier, *GADD *genes are known to be modified through HAT/HDAC balance [201], as well as ATM kinase activity [186,225].

Curcumin also inhibits DNA repair pathways in cancer cells, like the fanconi anemia/BRCA (FA/BRCA) pathway [226], or downregulates DNA repair proteins MGMT (O^6^-methylguanine-DNA methyltransferase), DNAPK, Ku70, Ku80, and ERCC-1 [227]. Other studies have shown that curcumin induces damage to both mitochondrial and nuclear DNA [228], triggers ROS generation [229] and glutathione (GSH) depletion [230], resulting in apoptosis induction in cancer cells.

#### Resveratrol

Resveratrol, a stilbene found in grapes and wine, has been implicated in anti-aging and cancer prevention mechanisms [231]. Resveratrol was linked with activation of SIRT1 and the acetyl transferase, p300 [94,232,233]. There is a debate as to whether these mechanisms are directly or indirectly involved in the protective effects of resveratrol in *vitro *and *in vivo *[234,235]. A recent study concluded that tumor suppressive effects of resveratrol in *Apc*^min ^mice were dependent on SIRT expression [236]. Resveratrol can delay cell cycle progression and induce apoptosis in several cancer cell lines; some of these effects have been attributed to the activity of SIRT1.

Several proteins that have a role in the DNA damage response, such as p53, FoxO, and Ku70, are deacetylated and inactivated by SIRT1. Consistent with this role of SIRT1, recent evidence indicates that resveratrol inhibits DNA repair in cancer cells [83,94,237-239]. Studies by Wang et al. [94] using SIRT1 mutant mice showed that impaired SIRT1 function resulted in tumor formation in a p53-null background, and that activation of SIRT1 by resveratrol reduced tumorigenesis. Further, SIRT1 activation by resveratrol negatively regulated survivin expression by histone deacetylation in the promoter of the *survivin *gene [237]. Resveratrol enhanced p53 acetylation and induced apoptosis in prostate cancer cells by inhibiting MTA1/NuRD, an integral component of the nucleosome remodeling and deacetylase complex [238]. Furthermore, resveratrol inhibited both HR and NHEJ via ATM-p53 and ATM/ATR-Nbs1-dependent pathways, respectively [239]. On the contrary, activation of SIRT1 by resveratrol was reported to promote APE1 activity and binding to X-ray cross-complementing-1 (XRCC1) protein, facilitating the BER DNA repair pathway [83].

Several studies support resveratrol inducing a sustained DNA damage response via BRCA1 and activation of the ATM/ATR-Chk1/2-Cdc25C pathway in cancer cells [240-242]. Notably, Tyagi et al [242] observed only marginal effects of resveratrol in normal human foreskin fibroblasts. In addition, a recent study demonstrated that resveratrol caused telomere instability in osteosarcoma cells [241], which resulted in genetic instability, activation of DNA damage response, and cell senescence.

There is increasing evidence that resveratrol exhibits "pro-oxidant" activity in some circumstances [231]. Resveratrol catalyzed oxidative DNA degradation in the presence of transition metal ions, such as copper [243], generated ROS [244,245], and triggered GSH efflux associated with Bax translocation to the mitochondria [246].

#### Isoflavones

Soy isoflavones have been implicated in reducing the overall incidence of breast and prostate cancers in Asian countries. Genistein (4',5,7-trihydroxyisoflavone) is the major isoflavone present in soybeans. Genistein is known to inhibit human cancer cell growth, mediated via genes controlling cell cycle progression and apoptosis [247]. One mechanism that has recently received considerable attention is the epigenetic modulation of DNA methylation and/or chromatin marks [248]. Genistein possesses high histone modifying activity compared with other isoflavones, such as biochanin A and diadzein. Genistein impacted histone acetylation and demethylation, leading to activation of tumor suppressors such as p21, p16, FoxO3a, and phosphatase and tensin homolog (PTEN) [249]. Genistein also caused androgen receptor (AR) downregulation through inhibition of HDAC6-Hsp90 co-chaperone functions in prostate cancer cells [250].

Genistein activated stress signaling pathways that phosphorylated p53 and ATM, leading to p21 induction and γH2AX formation [251,252]. Further, genistein modulated cyclin-dependent kinase Cdc2 activity through the protein phosphatase Cdc25C, thereby activating ATM and causing G_2_/M arrest in hepatoma cells [253]. Other recent studies confirmed similar effects in lung and prostate cancer cells [254-256]. An *in vivo *metabolite of genistein, 5,7,3',4'-tetrahydroxyisoflavone, was shown to act *via *ATR kinase signaling to cause DNA breaks and induce cell cycle arrest [257]. Genistein induced GADD45, p53, and p38 in embryonic cancer cells [258], and enhanced expression of BRCA1 [259] and MDC1 in neuroblastoma cells [260].

Other reported mechanisms for genistein in cancer cells include oxidative DNA damage by ROS generation in the presence of copper [261], and inhibition of topoisomerase II in an ATM-dependant manner [262]. Interestingly, in the non-cancerous MCF-10A breast cell line, genistein protected against polycyclic aromatic hydrocarbon (PAH)-induced oxidative DNA damage [263].

#### Quercetin

Quercetin is a flavonoid found in foods such as citrus fruit, buckwheat, and onions. Recently, quercetin was shown to increase histone H3 acetylation by both HAT activation and HDAC inhibition in leukemia HL60 cells. The result was FasL-dependent apoptosis, and activation of the extracellular signal-regulated kinase (ERK) and jun N-terminus kinase (JNK) signaling pathways [264]. Quercetin also induced the phosphorylation of ATM and H2AX [251]. Despite its anti-inflammatory and anti-oxidant properties, low concentrations of quercetin induced extensive DNA damage by reacting with Cu(II) in cancer cells [265]. This was confirmed recently, with evidence that a quercetin-copper(II) complex promoted cleavage of plasmid DNA, producing single and double DNA strand breaks in lung cancer A549 cancer cells [266]. In addition, quercetin inhibited DNA repair via competitive inhibition of DNAPK, a repair protein involved in NHEJ [267].

## Dietary compounds as chemo- and radio-sensitizers for cancer therapy

In addition to the aspects discussed above, histone modifiers exert synergistic actions when combined with ionizing irradiation (IR) or DNA-damaging drugs [268-270]. HDAC inhibitors can stabilize and enhance γH2AX and interfere with the DNA repair machinery in cancer cells [271]. From the evidence provided above, many dietary compounds can influence the DNA damage response and inhibit specific repair mechanisms. Importantly, histone modifications augment DNA damage in a manner that goes essentially unrepaired in many cancer cells, but is repaired effectively in normal cells. Some illustrative examples from the recent literature are provided below.

### ITCs, HDAC inhibitor drugs, and radiotherapies

Radiosensitivity of HeLa cells was reportedly enhanced by SFN pretreatment. Pre-treatment with SFN was found to inhibit DSB repair in irradiated cells leading to apoptosis. This was associated with decreased expression of repair proteins, Rad51 and DNAPK [272]. The authors showed that the combination was also effective *in vivo *[272]. In PC3 prostate cancer cells, the lipid peroxidation end-product 4-hydroxynonenal resulting from SFN treatment potentiated the anti-tumor effects of the HDAC inhibitor LBH589. Combined SFN+LBH589 treatment induced dephosphorylation of Cdc2 and sustained expression of γH2AX [273]. BITC and other ITCs sensitized pancreatic cancer cells to γ-irradiation. Specifically, BxPC-3 pancreatic cancer cells pretreated with 2.5 μM BITC for 24 h followed by exposure to 5 Gy γ-irradiation had reduced survival and enhanced G_2_/M arrest as compared to cells exposed to γ-irradiation alone. Cell cycle arrest was associated with DNA damage, phosphorylation of ATR, Chk2, Cdc25C, and Cdk-1, and induction of p21 [274]. Similarly, PEITC significantly enhanced cytotoxicity in a vorinostat-resistant leukemia cell line, HL60/LR, by inhibiting the cytoprotective antioxidant response involving depleted cellular GSH [275].

### Anacardic acid enhances radiosensitivity

HAT inhibitors in the anacardic acid family (see above) exert antiproliferative and cytotoxic effects on pituitary adenoma cells associated with an increase in PARP, sub-G_1 _arrest, and apoptosis. These compounds radiosensitized pituitary adenoma cells by reducing the expression of survivin and X-linked inhibitor of apoptosis protein (XIAP), which are known to be associated with cell survival and radioresistance [276].

### Curcumin synergizes with chemo- and radiotherapy

Curcumin has been shown to enhance the toxicity of cyclophosphamide (CTX) in a drug-resistant human lymphoma cell line HT/CTX, via inhibition of the FA/BRCA pathway. The combination of curcumin and CTX produced synergistic effects and reversed multiple drug resistance. Blockade of cell cycle progression and downregulation of fanconi anemia group D2 (FANCD2) were implicated in the anti-tumor mechanism of curcumin [277]. Similarly, curcumin reversed multidrug resistance in multiple myeloma cell line MOLP-2/R through inhibition of FA/BRCA, suggesting beneficial outcomes when used with low-dose DNA cross-linking agents [278]. In a variety of human cancer cells, synergistic inhibition of cell proliferation also was seen for curcumin combined with cisplatin, 5-fluorouracil (5-FU), or celecoxib, via inhibition of DNA repair pathways [226,279-281]. Curcumin sensitized glioma cells to clinically used chemotherapeutic agents or radiation, which correlated with reduced Bcl-2 and inhibitor of apoptosis (IAP) family members as well as DNA repair enzymes MGMT, DNAPK, Ku70, Ku80, and the excision repair cross-complementary-1 (ERCC-1) [227]. Recently, Lin et al [282] have shown that curcumin downregulates the expression levels of thymidine phosphorylase (TP), an enzyme of the pyrimidine salvage pathway and ERCC1, a protein involved in the process of nucleotide excision repair which helps in overcoming platinum resistance in cancer cells. Interestingly, curcumin also synergized with HDAC inhibitors vorinostat and LBH589, via persistent depletion of Hsp90 client proteins EGFR, Raf-1, Akt, and survivin [283].

### Resveratrol and purine analogs

In chronic lymphocytic leukemia (CLL) cells from patients, clinically-used purine analogs fludarabine or cladribine caused a higher rate of apoptosis when combined with resveratrol. Apoptosis was related to the presence of cytogenetic abnormalities and increased DNA damage markers γH2AX and ATM. The authors suggested that resveratrol might provide a new therapeutic approach for CLL due to acceptable safety, lowering the dose of purine analogs, resulting in activation of DNA damage specifically in cancer cells and not in normal cells [284].

### Catechins and COX-2 inhibitors

EGCG, in combination with COX-2 inhibitors enhanced apoptosis by increasing the expression of DNA damage- inducible *GADD153, GADD45A*, and *CDKN1A *(p21/WAF1/CIP1) genes. Synergistic enhancements of apoptosis and GADD153 gene expression in human non-small cell lung cancer cells by the combination of EGCG and celecoxib were mediated through the activation of the MAPK signaling pathway [199].

## *In vivo *studies and clinical translation: Future perspectives

*In vivo *studies that demonstrate the functional relevance of epigenetic mechanisms for anti-tumor efficacy are still relatively scarce. At present, the best evidence to demonstrate that nutrition modulates epigenetic status and health outcomes in mammals comes from studies with mice carrying the agouti (*Avy*) gene [285]. Dietary methyl deficiency (folate, choline, and methionine) in animal models alters hepatic DNA methylation patterns and induces liver cancer in the absence of a carcinogen [286]. Similarly, selenium-deficient diets have been shown to hypomethylate DNA in liver and colon, as compared to rats fed either selenite or selenomethionine [287]. In a very interesting study, high levels of grooming and nursing by rat mothers modified the levels of DNA methylation at a glucocorticoid receptor (GR) gene promoter in the hippocampus of the offspring, leading to altered histone acetylation and binding of a transcription factor (NGFI-A) to the GR promoter [288]. Further, it was observed that a proportion of these changes could be modified by treatment with an HDAC inhibitor or a methyl donor [289].

Dietary HDAC inhibition also is an emerging field, with some evidence for epigenetic modulation *in vivo*. For example, polyphenon B, a tea polyphenol preparation, decreased HDAC1 levels and modulated the expression of markers of invasion and angiogenesis in dimethylaminoazobenzene-induced liver cancer in rats [290]. Theophylline, also present in tea, was associated with downregulation of the inflammatory response through increased HDAC activity in epithelial cells and macrophages in smokers and chronic obstructive pulmonary disease (COPD) patients, a situation associated with diminished HDAC activity [291-294]. It is noteworthy that the mechanism occurred at therapeutic concentrations [294]. Another polyphenol, quercetin, inhibited HDAC1 and DNA methyl transferase 1 (DNMT1) in carcinogen-treated hamsters and reduced tumor incidence and burden [295].

In the ApcMin/+ mouse model, SFN-containing diet (300 and 600 ppm for 3 weeks) was optimal for achieving SFN tissue concentrations in the 3-30 μM range [296]. In the same animal model, we reported that SFN-containing diet suppressed tumor development via increased global H3/H4 histone acetylation, with a concomitant upregulation of *p21 *and *Bax *gene expression [297]. In another study, Myzak et al. [298] demonstrated that oral administration of 7.5 μM SFN per animal per day for 21 days significantly reduced growth of prostate cancer (PC-3) tumor xenografts and decreased HDAC activity in the xenografts, prostates, and mononuclear blood cells. There was a trend towards increased global histone acetylation in these tissues. The study was also extended to human volunteers wherein consumption of 68 g broccoli sprouts resulted in a significant inhibition of HDAC activity in peripheral blood mononuclear cells 3 h following intake [298]. Recently, it was demonstrated that SFN is highly metabolized in mice, achieving micromolar concentrations in plasma, with tissue concentrations in the range 0.003 - 0.35 nmole/mg. Thus, SFN metabolites may play an important role in HDAC and tumor inhibition [299]. *In vivo *data with BITC, another ITC, clearly indicate that oral administration of 12 μmol BITC significantly suppressed the growth of pancreatic (BxPC-3) tumor xenografts, and that tumor suppression was associated with the reduced NF-κB, cyclin D1, HDAC1, and HDAC3, complementing observations made *in vitro*. The authors suggested inhibition of HDAC1/HDAC3 by BITC as a plausible mechanism of NF-κB inactivation [124]. Other studies have shown that ITCs can achieve therapeutic serum concentrations both *in vivo *[300] and in humans [301].

In rats, treatment with AM and/or DADS increased acetylation of histones and caused up-regulation of p21 expression in normal liver and hepatoma cells and in rat colonocytes [163,302,303]. However, there is concern about the high concentration of allyl-derivatives used in these studies, which may be associated with toxicity in various tissues.

Li et al. [150] provided direct *in vivo *evidence on the role of dietary HDAC inhibition and DNA damage for the anti-cancer effects of DIM, an I3C metabolite. Previous reports by Bhatnagar et al. [149] showed DIM significantly inhibited expression of HDAC1, HDAC2 and HDAC3 in colon cancer cells and in APCmin/+ mice. Li et al. [150] demonstrated using colon cancer (HT29) xenografts that DIM downregulates HDAC1 and HDAC2 and this was associated with induction of γH2AX and p21 expression in the xenografts. Importantly, these effects were seen at non-toxic DIM concentrations. An oral dose of 250 mg/kg of DIM produced a plasma concentration of 18 μg/ml in mice, equivalent to ~77 μM. The authors suggested DNA damage as a possible mechanism of cancer cell death induced by DIM [150].

*In vivo *studies with dietary polyphenols have shown encouraging results on DNA damage and tumor inhibition. Tyagi et al [304] showed that resveratrol (50 mg/kg bw) treatment inhibited head and neck squamous cell carcinoma (FaDu) tumor growth in nude mice, and γH2AX and cleaved caspase-3 were strongly increased in xenografts from resveratrol-treated mice compared to controls. Vanhees et al. [305] have shown that prenatal exposure to both genistein and quercetin supplements in mice induced DSBs and DNA rearrangements in the *mixed-lineage leukemia *(*MLL*) gene, especially in the presence of compromised DNA repair. Toyoizumi et al. [306] reported that co-administration of isoflavones and NaNO_2 _caused DNA damage in mouse stomach via the formation of radicals. Amin et al. [307] observed that EGCG, in combination with luteolin, increased apoptosis in head and neck and lung cancer xenografted tumors in nude mice, possibly by ATM-dependent Ser(15) phosphorylation of p53 resulting from DNA damage.

Pharmacological HDAC inhibitors also have shown promise when acting *in vivo*, alone or in combination with radiotherapy/chemotherapy. Vorinostat, as a single agent, was shown to induce DSBs associated with the downregulation of DNA repair gene *Rad52*, thus preventing brain metastasis of triple-negative breast cancer [115]. In a murine metastatic neuroblastoma model, vorinostat was found effective possibly by modulating DNA repair enzyme Ku-86 [308]. Treatment with LBH589, another HDAC inhibitor, led to a dramatic reduction of tumor growth in a colon (HCT116) cancer model. Analysis of the residual tumor revealed that HDAC inhibitor treatment increased histone acetylation, γH2AX accumulation, and apoptosis. The treatment had no obvious detrimental effects on the mice, only acting on the xenografts [309]. In addition, various HDAC inhibitors appear to sensitize tumors to IR *in vivo*, as demonstrated by vorinostat [308,310], MS-275 [311], valproic acid [312], LBH589 [313], LAQ824 [314], AN-9 [315] and PCI-24781 [316]. Treatment with these HDAC inhibitors led to greater delay in tumor growth by enhancing IR-induced γH2AX in xenografts, suggesting that HDAC inhibitors interfere with DSB repair and/or render DNA more susceptible to IR-induced damage. However, there is evidence that radiosensitization is not limited to cancer cells, but also occurs in healthy normal cells. Vorinostat, MS-275, sodium-butyrate and valproic acid treatments have been shown to increase radiosensitivity and reduce DSB repair capacity in normal cells too, leading to potential genotoxic effects of HDAC inhibitor treatment [317].

In addition to the growing list of studies *in vivo*, there are over 300 human clinical trials of HDAC inhibitors, tested either alone or in combination with radiation, chemotherapy, and/or molecular therapy. In particular, some of the human trials with vorinostat and valproic acid in combination with radiation are evaluating the effects on DNA damage and repair factors http://clinicaltrials.gov/. While *in vitro *models have contributed enormously to our mechanistic understanding of the epigenetic network and its regulation, there remains a paucity of preclinical and clinical data for the majority of dietary compounds. Until this situation is rectified, one must exercise caution when interpreting and extrapolating the significance of current evidence in the literature. Ongoing clinical trials are moving in the right direction, as for example in the evaluation of broccoli sprouts and broccoli sprout extract for modulating epigenetic marks in breast and prostate cancer. In addition to testing for epigenetic biomarkers in blood and tumor biopsies, DNA damage markers (e.g. γH2AX) could be analyzed in tumor samples and adjacent normal tissue to provide insights on the DNA damage response. Monitoring γH2AX levels in a patient's circulating tumor cells [318], PBMCs, or hair samples might prove useful in the clinical setting [319,320]. Studies in (normal) human volunteers could certainly benefit from such non-invasive techniques. However, it will be important to note that tumor cells also have genetic alterations that impact responses to DNA damage, which differ from normal replicating cells. Notably, normal cells typically respond in a facile manner to "correct' DNA damage responses once the test agent has been removed.

## Conclusions

**Figure 3:**
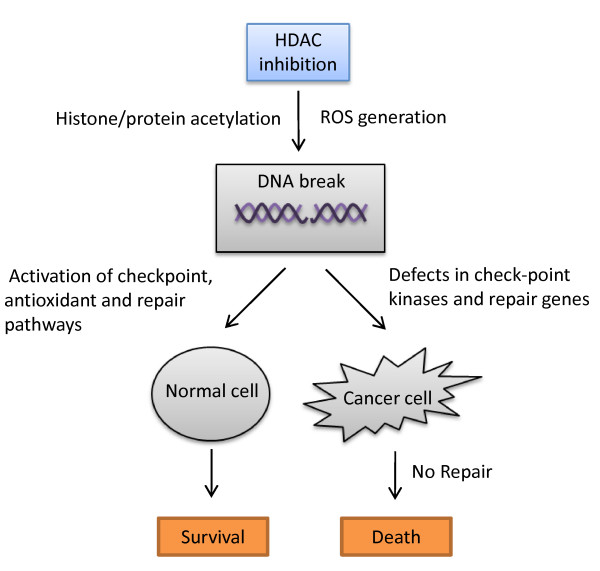
**The differential effect of DNA damaging agents in cancer and normal cells**. HDAC inhibitors are known to cause DSBs through chromatin remodeling and oxidative damage due to ROS generation. Normal cells counteract this by check point activation leading to cell cycle arrest; anti-oxidant mechanisms and effective DNA repair whereas cancer cells known to be defective in some of these mechanisms, for e.g. check point kinases and repair genes, fail to repair the DNA damage leading to cell death.

Genomic instability provides a means for selective targeting of cancer cells over normal cells, via epigenetic players with important roles in DNA repair. The active recruitment and de-recruitment of HAT and HDAC enzymes and their binding partners at sites of DNA damage produces localized sites of open chromatin, increasing the genotoxic effectiveness of agents such as UV, IR, and chemotherapeutic agents. The literature supports the role of multiple HDACs in genome surveillance, and HDAC inhibitors appear to facilitate cancer cell death by enhancing the DNA damage response and inhibiting DNA repair. Among the various cancer chemopreventive agents reviewed herein, many cause changes in chromatin conformation, disrupt the intracellular redox balance, and deregulate DNA repair proteins. Thus, these compounds might activate the DNA damage response with particular effectiveness in cancer cells as compared to normal cells as depicted in Figure 3. *In vivo *therapeutic efficacy of these compounds, as reviewed here and elsewhere [29], suggests that effective concentrations are achievable and modulate DNA damage and repair responses in tumors. Dietary compounds with pleiotropic effects in cancer cells also likely impact DNA damage and repair via other epigenetic mechanisms, such as through DNA methylation and microRNAs. Improved understanding of these various epigenetic mechanisms will, it is hoped, provide a more rational basis for combining specific dietary compounds and standard radiation or chemotherapy approaches, thereby enhancing efficacy in the clinical setting.

## Abbreviations

53BP1: p53-binding protein; 5-FU: 5-fluorouracil; 6-PDSA: 6-pentadecyl salicylic acid; allyl-ITC: allyl isothiocyanate; AM: allyl mercaptan; AMS: allyl methyl sulfide; APE1: apurinic/apyrimidinic endonuclease-1; APE1/Ref-1: apurinic apyrimidinic endonuclease redox effector factor-1; Asf1: anti-silencing function 1; ATM: ataxia-telangiectasia mutated; ATR: ATM-and Rad3-related; ATX: ATM related kinase; BER: base-excision repair; BITC: benzyl isothiocyanate; BLM: bloom syndrome gene; BRCA1: breast cancer 1; BRCA2: breast cancer 2; CAF1: chromatin assembly factor I; CHK1/CHK2: checkpoint kinase1/2; CK2: casein kinase 2; CLL: chronic lymphocytic leukemia; CtIP: c-terminal binding protein interacting protein; CTX: cyclophosphamide; DADS: diallyl disulfide; DAS: diallyl sulfide; DATS: diallyl trisulfide; DIM: 3,3'-diindolyl methane; DMBA: 7: 12-dimethylbenz[a]anthracene; DNAPK: DNA-dependent protein kinase; DSB: double-strand break; EGCG: (-)-epigallocatechin-3-gallate; ERK: extracellular signal-regulated kinase; FA/BRCA: fanconi anemia/BRCA pathway; FANCD2: fanconi anemia group D2; FoxO: forkhead box O; GADD153: growth arrest and DNA damage 153; GADD45: growth arrest and DNA damage 45; GSH: glutathione; H_2_O_2_: hydrogen peroxide; HAT: histone acetyl transferase; HDAC: histone deacetylase; HMGN1: high-mobility group N1; HP1β: heterochromatin Protein 1β; HR: homologous recombination; I3C: indole-3-carbinol; IAP: inhibitor of apoptosis; Idh2: isocitrate dehydrogenase 2; IL6: interleukin 6; ING1a: inhibitor of growth 1a; IR: ionizing irradiation; ITC: isothiocyanate; JNK: jun N-terminus kinase; KAP-1: KRAB associated protein; KMSB: keto-methylselenobutyrate; Ku70: nonhomologous end joining (NHEJ) factor; LBH589: panobinostat; MCL1: induced myeloid leukemia cell differentiation protein; MDC1: mediator of DNA damage checkpoint protein-1; Mdm2: murine double minute; MEFs: murine embryonic fibroblasts; MGMT: O^6^-methylguanine-DNA methyl transferase; MLH1: MutL homolog 1; MMR: mismatch repair; MRE11: meiotic recombination 11; MRN: MRE11-RAD50-Nbs1 complex; MSA: methylseleninic acid; MSC: methyl selenocysteine; MSP: methyl selenopyruvate; NAC: N-acetyl cysteine; NADPH: nicotinamide adenine dinucleotide phosphate; Nbs: nijmegen breakage syndrome; NCOR: nuclear receptor corepressor; NER: nucleotide-excision repair; NFκB: nuclear factor kappa B; NHEJ: non-homologous end-joining; PARP: poly(ADP-ribose)polymerase; PcG: polycomb group protein; PCNA: proliferating cell nuclear antigen; PEITC: phenethyl isothiocyanate; PHITC: phenylhexyl isothiocyanate; PI3K: phosphatidylinositol-3 kinase; PN: parthenolide; PTEN: phosphatase and tensin homolog; RAD51: DNA repair protein RecA homolog; RelA: transcription factor p65; RFC: replication factor C; RNF8/168: RING finger E3 ubiquitin-protein ligase; ROS: reactive oxygen species; RPA: replication protein A; SAC: *S*-allylcysteine; SAHA: suberoylanilide hydroxamic acid; SAMC: *S*-allylmercaptocysteine; SFN: sulforaphane; Sir2: silent information regulator 2; SIRT: sirtuin; SM: selenomethionine; SMRT: silencing mediator for retinoic and thyroid receptor; SSA: single-strand annealing; SSB: single-strand break; SWI2/SNF2: SWItch/Sucrose NonFermentable; Tip60: TAT-interacting protein 60; TP: thymidine phosphorylase; TrxR: thioredoxin reductase; VPA: valproic acid; WRN: werner helicase; XIAP: X-linked inhibitor of apoptosis protein; XPA: xeroderma pigmentosum group A; XPC: xeroderma pigmentosum group C; XRCC1: X-ray cross-complementing-1; γH2AX: phosphorylated histone H2AX.

## Competing interests

The authors declare that they have no competing interests.

## Authors' contributions

PR wrote the first draft of the article. EH, DEW and RHD edited and finalized the manuscript. All authors read and approved the final manuscript.
